# Supplementary material for: TET2 lesions enhance the aggressiveness of CEBPA-mutant acute myeloid leukemia by rebalancing GATA2 expression
Source: Nat Commun. 2023 Oct 4;14:6185. doi: 10.1038/s41467-023-41927-x (PMC10550934; doi:10.1038/s41467-023-41927-x)
Supplement: Supplementary file 1 — Supplementary Information [file 41467_2023_41927_MOESM1_ESM.pdf]

SUPPLEMENTAL INFORMATION

TET2 lesions enhance the aggressiveness of *CEBPA*-mutant acute myeloid leukemia by rebalancing *GATA2* expression

Elizabeth Heyes[†,1], Anna S. Wilhelmson[†,2,3,4], Anne Wenzel[2,3,4], Gabriele Manhart[1], Thomas Eder[1], Mikkel B. Schuster[2,3,4], Edwin Rzepa[1], Sachin Pundhir[2,3,4], Teresa D'Altri[2,3,4], Anne-Katrine Frank[2,3,4], Coline Gentil[2,3,4], Jakob Woessmann[5], Erwin M. Schoof[5], Manja Meggendorfer[6], Jürg Schwaller[7], Torsten Haferlach[6], Florian Grebien[‡,1,8], and Bo T. Porse[‡,2,3,4,9]

[†]Co-first author
[‡]Co-senior author, co-corresponding author

[1]University of Veterinary Medicine, Institute of Medical Biochemistry, Vienna, Austria
[2]The Finsen Laboratory, Copenhagen University Hospital - Rigshospitalet, Copenhagen, Denmark
[3]Biotech Research and Innovation Centre (BRIC), Faculty of Health Sciences, University of Copenhagen, Copenhagen, Denmark
[4]Danish Stem Cell Center (DanStem) Faculty of Health Sciences, University of Copenhagen, Copenhagen, Denmark
[5]Department of Biotechnology and Biomedicine, Technical University of Denmark, Lyngby, Denmark
[6]MLL Munich Leukemia Laboratory, Munich, Germany.
[7]Department of Biomedicine, University Children's Hospital Basel, Basel, Switzerland
[8]St. Anna Children's Cancer Research Institute (CCRI), Vienna, Austria
[9]Department of Clinical Medicine, University of Copenhagen, Copenhagen, Denmark

SUPPLEMENTAL FIGURES

Supplemental figure 1, related to Figure 1:

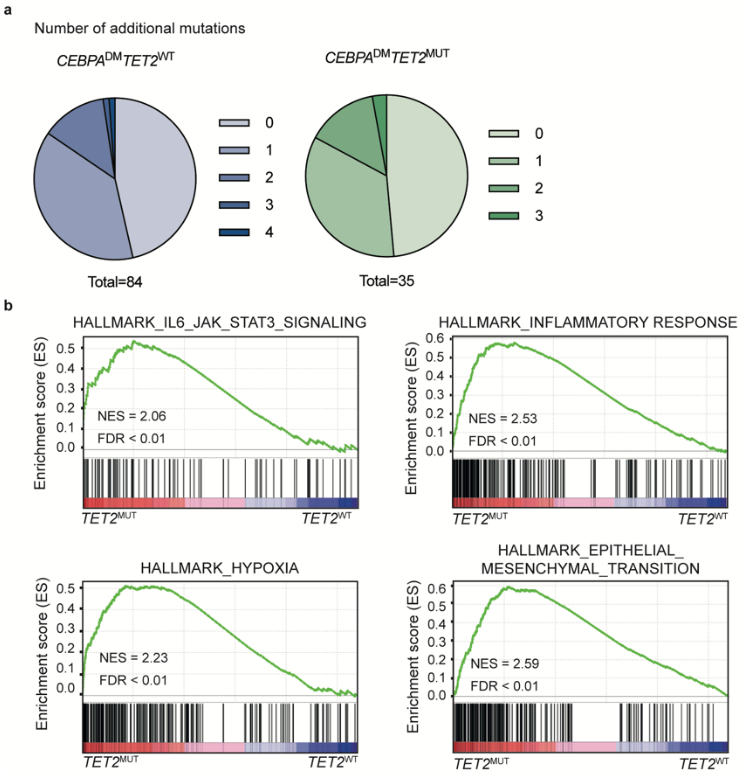

(**a**) Number of additionally accumulated mutations in *CEBPA*^{DM}*TET2*^{WT} and *CEBPA*^{DM}*TET2*^{MUT} patients (*TET2*^{WT} 84 and *TET2*^{MUT} 35 patients). (**b**) Gene set enrichment analysis (GSEA) of the *TET2*^{WT} versus *TET2*^{MUT} *CEBPA*-mutant patients in the Beat AML dataset (*TET2*^{WT} 11 and *TET2*^{MUT} 5 patients).
Source data are provided as a Source Data file.
NES=normalized enrichment score; FDR=false discovery rate

Supplemental figure 2, related to Figure 2:

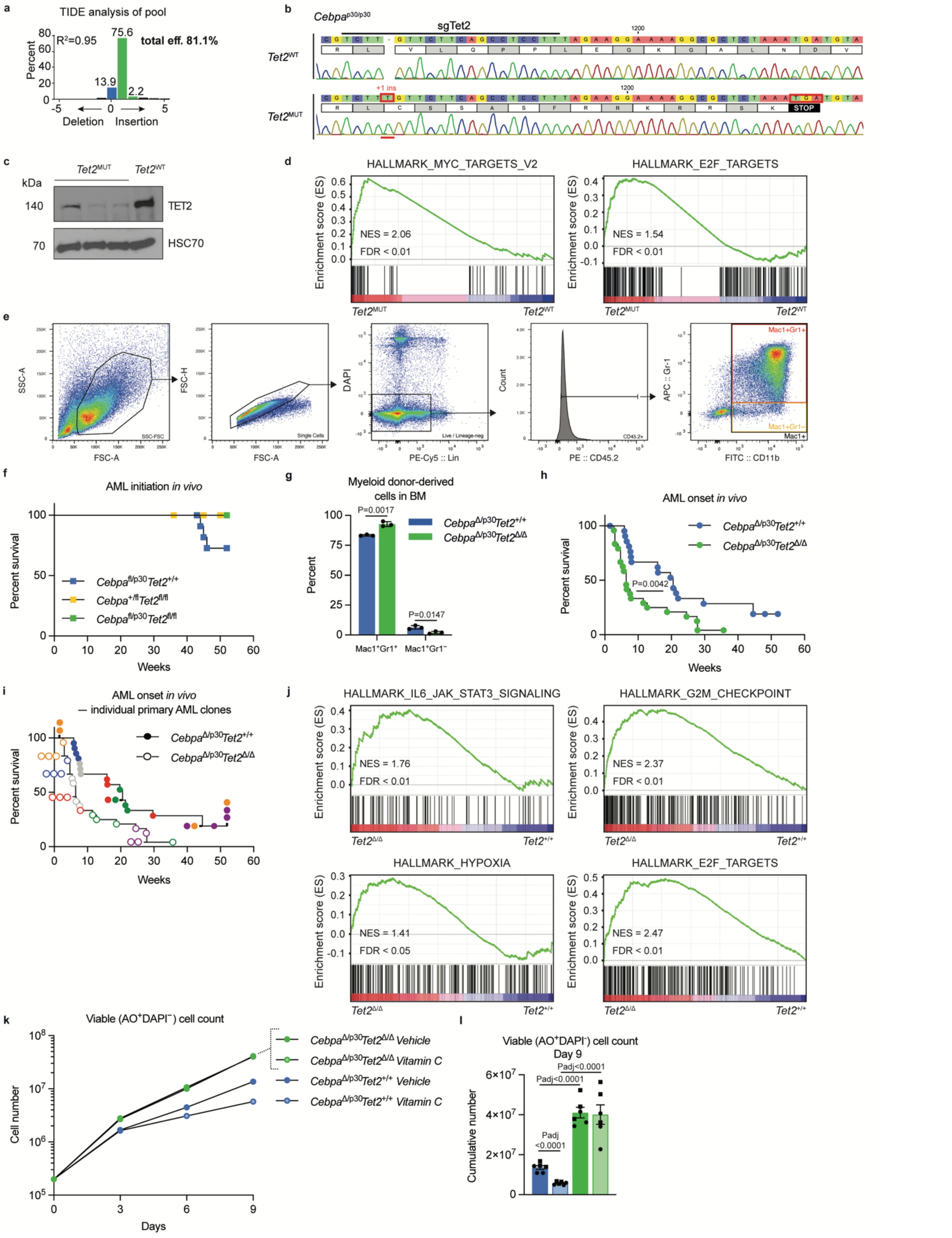

(**a**) Tracking of indels by decomposition (TIDE) of a mixed pool of *Cebpa*$^{p30/p30}$ cells 3 months post CRISPR-targeting of *Tet2*. (**b**) Sanger sequencing analysis of the mutated *Tet2* region in a *Cebpa*$^{p30/p30}$*Tet2*^{WT} and a *Cebpa*$^{p30/p30}$*Tet2*^{MUT} clone

(showing 1 representative clone of 9 clones with +1 inserts). (**c**) Western blot showing TET2 protein levels in 3 *Cebpa*$^{p30/p30}$*Tet2*^{MUT} clones compared to *Cebpa*$^{p30/p30}$*Tet2*^{WT}. (**d**) Gene set enrichment analysis (GSEA) plots for selected upregulated gene sets of *Cebpa*$^{p30/p30}$*Tet2*^{MUT} versus *Cebpa*$^{p30/p30}$*Tet2*^{WT} (*Tet2*^{WT} 7 and *Tet2*^{MUT} 5 clones). (**e**) Sorting and analysis strategy for *in vivo* leukemic blasts. (**f**) AML initiation after transplantation and Cre-LoxP recombination of control mice not carrying *Mx1-Cre* (*Cebpa*$^{fl/p30}$*Tet2*$^{+/+}$ 12, *Cebpa*$^{fl/+}$*Tet2*$^{fl/fl}$ 13, and *Cebpa*$^{fl/p30}$*Tet2*$^{fl/fl}$ 7 and mice). (**g**) Myeloid markers in donor-derived cells in bone marrow from moribund mice assessed by flow cytometry (3 mice per genotype). Data are presented as mean±SEM and analyzed by two-tailed unpaired t-test. (**h**) Survival of lethally irradiated secondary recipient mice after transplantation of leukemic BM from moribund primary recipient mice together with normal BM cells (*Cebpa*$^{\Delta/p30}$*Tet2*$^{+/+}$ 23 and *Cebpa*$^{\Delta/p30}$*Tet2*$^{\Delta/\Delta}$ 24 mice). The data were analyzed by Mantel-Cox Log-rank test. (**i**) Survival of secondary recipient mice with primary AML clone indicated by color (6 primary AML clones per genotype; 3 or 4 secondary recipient mice per primary AML). (**j**) GSEA plots for selected upregulated gene sets of *Cebpa*$^{\Delta/p30}$*Tet2*$^{\Delta/\Delta}$ versus *Cebpa*$^{\Delta/p30}$ *Tet2*$^{+/+}$ (sorted blasts from 3 mice per genotype). (**k**) Cell growth *ex vivo* assessed in the presence of Vitamin C or vehicle shown as viable (AO^{+}DAPI^{-}) cell count and (**l**) cumulative cell number after 9 days. The experiments were run with a total of 2 biological replicates per genotype (performed on separate days) where each experiment assayed 1 line per genotype. Each condition (Vitamin C and vehicle) was performed in technical triplicates for each of the 2 biological replicates per genotype. Data are presented as mean±SEM and the individual cell lines are indicated by circles or squares. The data was log-transformed and analyzed by two-way-ANOVA followed by Šídák correction for multiple comparison. Source data are provided as a Source Data file.
NES=normalized enrichment score; FDR=false discovery rate

Supplemental figure 3, related to Figure 3:

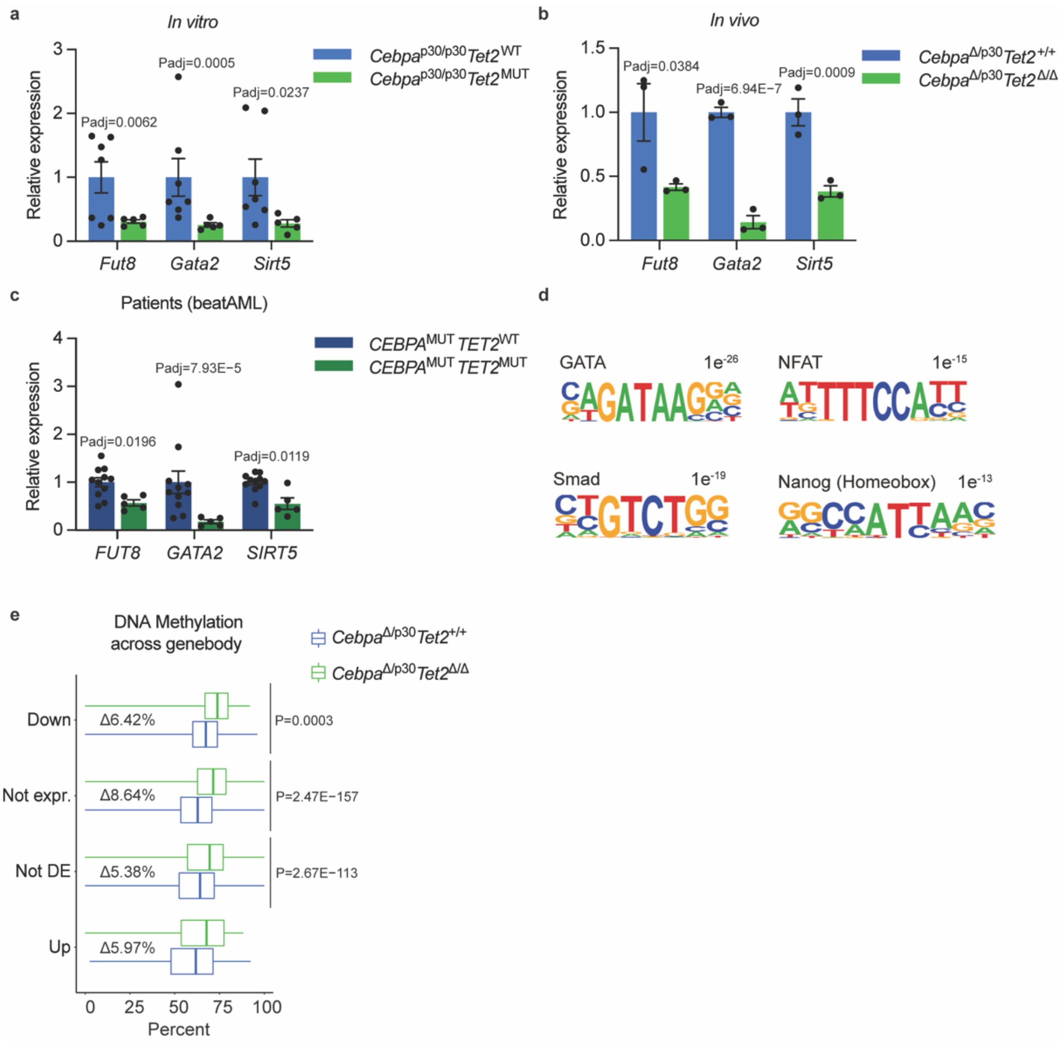

(**a**) Target RNA levels upon TET2-deficiency in *Cebpa*$^{p30/p30}$ cells (*Tet2*^{WT} 7 and *Tet2*^{MUT} 5 clones), and (**b**) *Cebpa*$^{\Delta/p30}$ leukemic blasts (sorted blasts from 3 mice per genotype), as well as (**c**) *CEBPA*^{MUT}*TET2*^{MUT} vs. *CEBPA*^{MUT}*TET2*^{WT} cases from the Beat AML dataset (*TET2*^{WT} 11 and *TET2*^{MUT} 5 patients). Data are presented as mean±SEM. Differential analysis was performed with DESeq2. (**d**) Motifs enriched in differentially accessible promoter regions upon *Tet2* mutation in *Cebpa*$^{p30/p30}$ cells (4 clones per group). Motif enrichment analysis was performed with HOMER. (**e**) Percent DNA methylation (mC) in sorted AML blasts across the gene bodies of down- (n=176), not expressed (n=6996), not differentially expressed (not DE; n=14816) and up-regulated (n=58) genes (samples from 2 *Cebpa*$^{\Delta/p30}$*Tet2*$^{+/+}$ and 3 *Cebpa*$^{\Delta/p30}$*Tet2*$^{\Delta/\Delta}$ mice). Boxes indicates the lower quartile-median-upper quartile and whiskers max–min. Data were analyzed by two-tailed unpaired t-test.

Source data are provided as a Source Data file.

Supplemental figure 4, related to Figure 4:

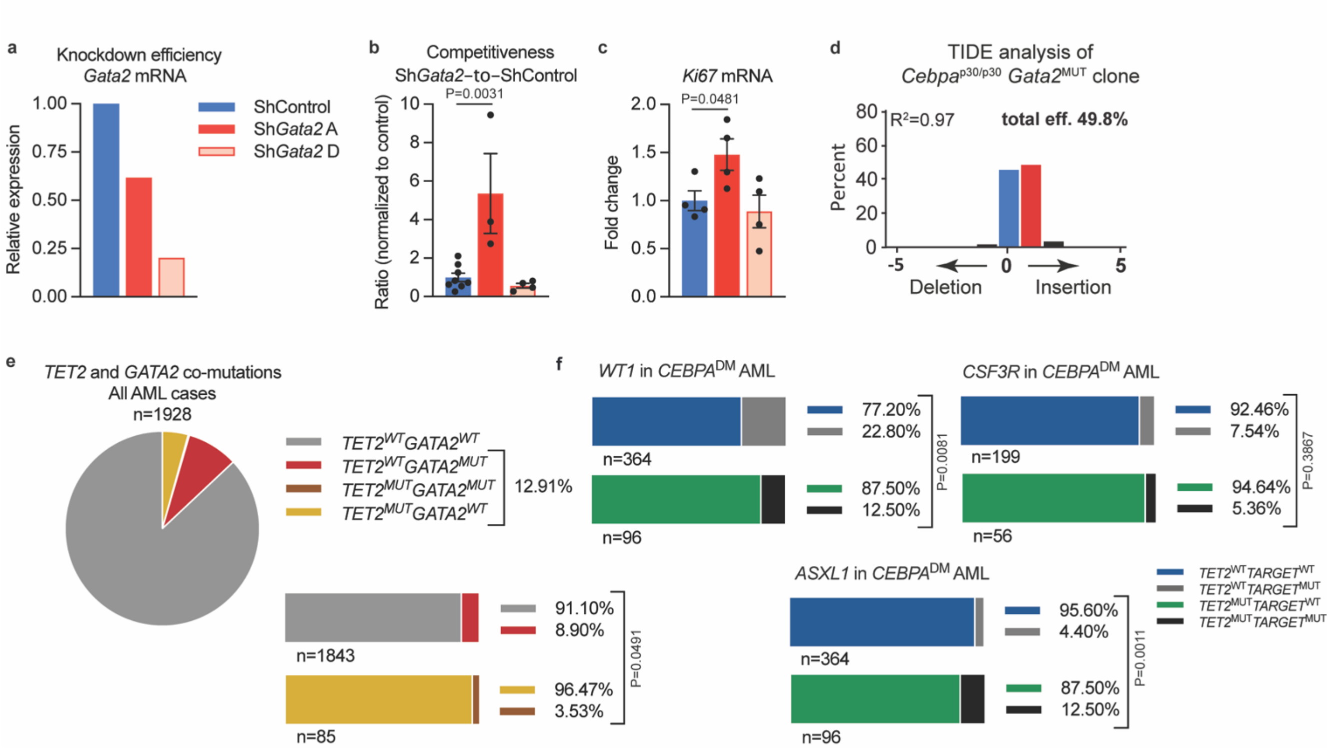

(**a**) *Gata2* mRNA levels in shRNA transduced *Cebpa*$^{p30/p30}$ leukemic cells prior to transplantation and (**b**) *in vivo* Sh*Gata2*-to-ShControl cell ratio normalized to control at 28 days after transplantation of the cells into sub-lethally irradiated recipients (4 mice per group). Data are presented as mean±SEM. Data were log-transformed and analyzed by one-way-ANOVA followed by Dunnett's multiple comparisons correction. (**c**) Ki67 expression in sorted sh*Gata2* A- and D-expressing cells relative to shControl-expressing cells (sorted cells from 4 mice per group). Data are presented as mean±SEM and analyzed by one-way-ANOVA followed by Dunnett's multiple comparisons correction. (**d**) Tracking of indels by decomposition (TIDE) analysis of a *Cebpa*$^{p30/p30}$*Gata2*^{MUT} clone (showing 1 representative clone of 10 clones with +1 inserts). (**e**) Presence or absence of *GATA2* mutations (*GATA2*^{MUT}) in all AML cases with or without *TET2* mutations (*TET2*^{MUT}) (detailed in Supplemental table 2b). (**f**) Presence or absence of *WT1, CSF3R,* and *ASXL1* mutations in *CEBPA*^{DM} AML cases with or without *TET2*^{MUT} (detailed in Supplemental tables 2c–e). Data were analyzed by Wilson/Brown binominal test.
Source data are provided as a Source Data file.

Supplemental figure 5, related to Figure 5:

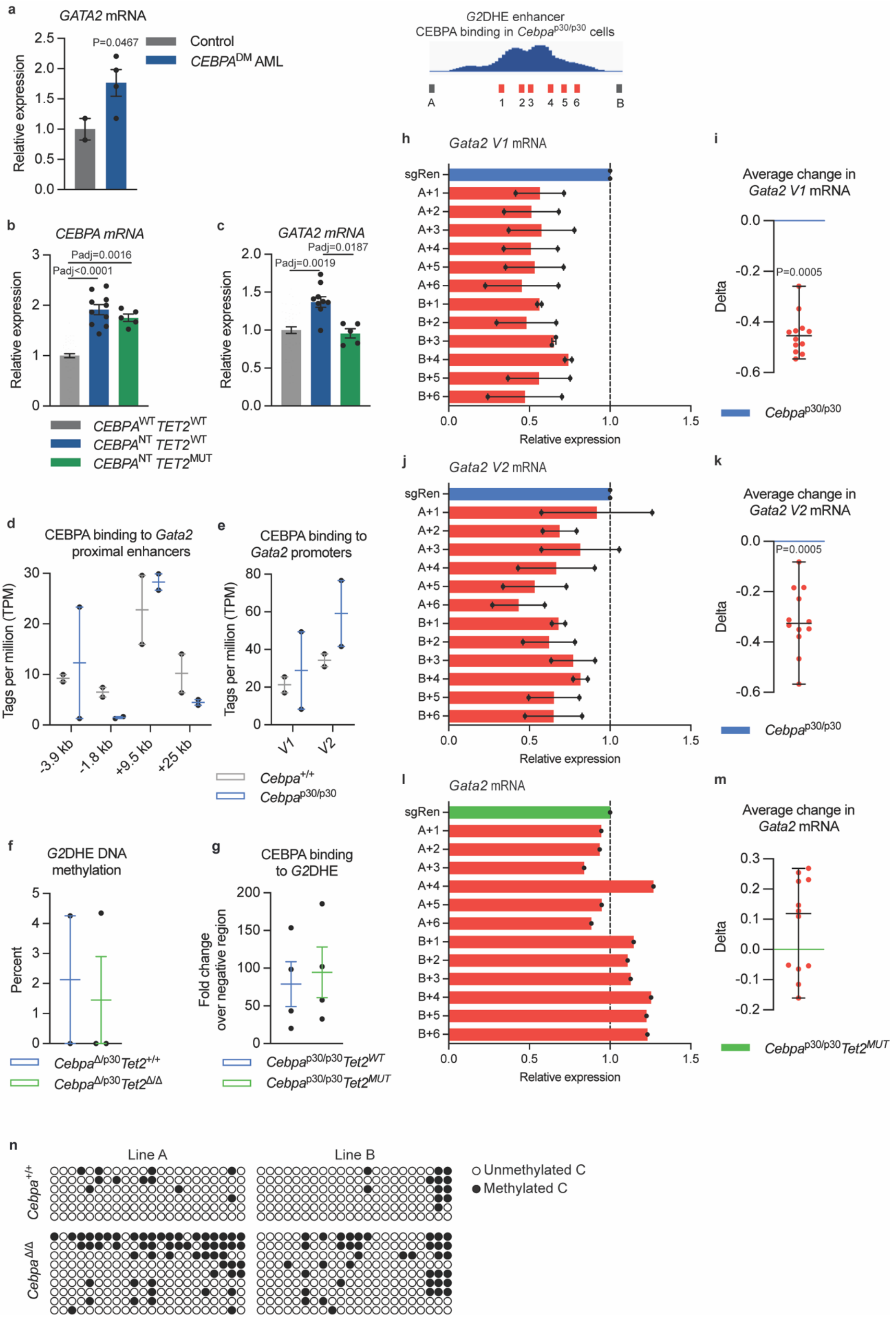

(**a**) *GATA2* mRNA expression in human *CEBPA*^{DM} AML vs. normal hematopoietic cells (BM samples from 4 *CEBPA*^{MUT} patients and 2 healthy controls), data from Jakobsen et al.[1]. Data are presented as mean±SEM. Differential analysis was performed with DESeq2. (**b**) *CEBPA* mRNA and (**c**) *GATA2* mRNA expression in human *CEBPA*^{NT} AML with or without

TET2^{MUT} vs. *CEBPA*^{WT}*TET2*^{WT} AML (*CEBPA*^{WT}*TET2*^{WT} n=52, *CEBPA*^{NT}*TET2*^{WT} n=10, and *CEBPA*^{NT}*TET2*^{MUT} n=5) from the Beat AML dataset. Data are presented as mean±SEM and were analyzed by Kruskal-Wallis test followed by Dunn's correction for multiple comparisons. (**d**) CEBPA-binding at *Gata2* enhancer, and (**e**) promoter regions in mouse *Cebpa*$^{\mathrm{p30/p30}}$ leukemic granulocyte/monocyte progenitors (GMPs) vs. normal GMPs (samples from 2 mice per group), data from Jakobsen et al.[1]. Data are presented as mean±SEM. (**f**) *G2*DHE DNA methylation in *Cebpa*$^{\Delta/\mathrm{p30}}$*Tet2*$^{\Delta/\Delta}$ and *Cebpa*$^{\Delta/\mathrm{p30}}$*Tet2*$^{+/+}$ blasts (samples from 2 *Cebpa*$^{\Delta/\mathrm{p30}}$*Tet2*$^{+/+}$ and 3 *Cebpa*$^{\Delta/\mathrm{p30}}$*Tet2*$^{\Delta/\Delta}$ mice). Data are presented as mean±SEM. (**g**) CEBPA binding at *G2*DHE in *Cebpa*$^{\mathrm{p30/p30}}$*Tet2*^{WT} and *Cebpa*$^{\mathrm{p30/p30}}$*Tet2*^{MUT} cells (4 clones per group). Data are presented as mean±SEM. (**h+j**) *Gata2* variant mRNA in response to targeting of the *G2*DHE by dual-guided CRISPR-Cas9 in *Cebpa*$^{\mathrm{p30/p30}}$ cells *in vitro* using indicated sgRNAs and (**i+k**) the averaged change in *Gata2* variant mRNA levels of the 12 deletions (averaged data from 2 separate experiments). Data are presented as median±range and analyzed by two-tailed Wilcoxon signed-rank test. (**l**) *Gata2* mRNA in response to targeting of the *G2*DHE by dual-guided CRISPR-Cas9 in *Cebpa*$^{\mathrm{p30/p30}}$*Tet2*^{MUT} cells *in vitro* using indicated sgRNAs and (**m**) the averaged change in *Gata2* mRNA levels of the 12 deletions (data from 1 experiment). Data are presented as mean±range. (**n**) Schematic overview of CpG methylation in the *Gata2 V2* promoter upon *Cebpa* knockout in two lines of *MLL-AF9* leukemia (generated from GMPs from BM of 2 separate mice).
Source data are provided as a Source Data file.

Supplemental figure 6, related to Figure 6:

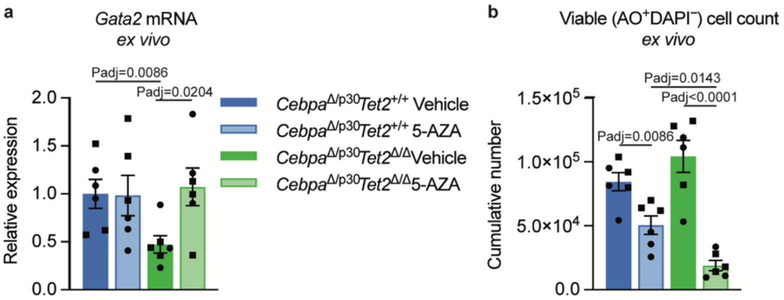

Ex vivo culture of leukemic cell lines in response to demethylating agent 5-Azacytidine (5-AZA) or vehicle. (**a**) *Gata2* mRNA expression, and (**b**) cell growth, measured as viable ($AO^{+}DAPI^{-}$) cell count, assessed after 6 days in the presence of 5-AZA or vehicle. The experiments were run with a total of 2 biological replicates per genotype (performed on separate days) where each experiment assayed 1 leukemic line per genotype. Each condition (5-AZA and vehicle) was performed in technical triplicates for each of the 2 biological replicates per genotype. Data are presented as mean±SEM and the individual cell lines are indicated by circles or squares. The data were analyzed by two-way-ANOVA followed by Šídák correction for multiple comparison.
Source data are provided as a Source Data file.

SUPPLEMENTAL TABLES

Supplemental table 1: Co-occurring mutations in *CEBPA*^{DM} AML cases

Cohort	*CEBPA*^{DM} (n)	*GATA2*	*TET2*	*WT1*	*NRAS*	*FLT3*	*CFSR3*	*DNMT3A*	*KIT*	*IDH1/2*	*ASXL1*	*EZH2*	*RUNX1*	*KRAS*	*SRSF2*	*NPM1*	*PTPN11*
Taube et al.[2], Blood, 2022	**131**	35%	24%	18%	14%	13%	2%	10%	5%	4%	4%	5%	3%	2%	2%	3%	0%
Zhang et al.[3], Cancer Gene Ther, 2019	**76**	32%	5%	30%	21%	16%	13%	0%	11%	1%	0%	7%	1%	3%	0%	1%	1%
Su et al.[4], Oncotarget, 2018	**81**	16%	12%	18%	15%	10%*	20%	5%	5%	8%	2%	9%	N.D.	N.D.	4%	2%	N.D.
Konstandin et al.[5], Blood Adv, 2018	**48**	35%	42%	30%	15%	23%	13%	15%	2%	0%	6%	4%	2%	2%	8%	0%	0%
Ahn et al.[6], Ann Hematol, 2016	**51**	14%	8%	14%	8%	8%*	N.D.	6%	N.D.	14%	4%	N.D.	N.D.	N.D.	N.D.	4%	N.D.
Papaemmanuil et al.[7], N Engl J Med, 2016	**66**	21%	9%	21%	27%	19%	N.D.	8%	6%	6%	0%	2%	0%	5%	0%	3%	5%
Fasan et al. [8], Leukemia, 2014	**104**	21%	35%	14%	N.D.	3%	N.D.	N.D.	N.D.	7%	14%	N.D.	6%	N.D.	N.D.	2%	N.D.
**Overall (%)** (mutated/all)	**100%** **557/557**	**26%** 142/555	**20%** 107/542	**19%** 103/556	**17%** 75/453	**12%** 67/557	**10%** 34/336	**7%** 32/453	**6%** 23/402	**5%** 30/557	**5%** 26/557	**5%** 21/402	**3%** 12/425	**3%** 9/321	**2%** 10/402	**2%** 13/557	**1%** 4/321

N.D. not determined; * *FLT3*^{TKD} only, *FLT3*^{ITD} N.D.

Supplemental table 2a: Overlap of *TET2*^{MUT} with *GATA2*^{MUT} in *CEBPA*^{DM} AML cases

Cohort	*CEBPA*^{DM} cases (n)	*TET2*^{WT} *GATA2*^{WT} (n)	*TET2*^{WT} *GATA2*^{MUT} (n)	*TET2*^{MUT} *GATA2*^{WT} (n)	*TET2*^{MUT} *GATA2*^{MUT} (n)
Taube et al.[2], Blood, 2022	131	61	38	24	8
Zhang et al.[3], Cancer Gene Ther, 2019	76	49	23	3	1
Konstandin et al.[5], Blood Adv, 2018	48	15	13	16	4
Ahn et al.[6], Ann Hematol, 2016	50	39	7	4	0
Papaemmanuil et al.[7], N Engl J Med, 2016	66	48	13	4	1
Fasan et al.[8], Leukemia, 2014	89	44	14	27	4
**Total (n)**	**460**	**256**	**108**	**78**	**18**
***TET2*^{WT} cases (n) (%)**	**364**	**256 (70.3%)**	**108 (29.7%)**	–	–
***TET2*^{MUT} cases (n) (%)**	**96**	–	–	**78 (81.3%)***	**18 (18.8%)***

* P=0.0103 (one-tailed Wilson/Brown Binominal test)

Supplemental table 2b: Overlap of *TET2*^{MUT} with *GATA2*^{MUT} in all AML cases

Cohort	All cases (n)	*TET2*^{WT} *GATA2*^{WT} (n)	*TET2*^{WT} *GATA2*^{MUT} (n)	*TET2*^{MUT} *GATA2*^{WT} (n)	*TET2*^{MUT} *GATA2*^{MUT} (n)
Ahn et al.[6], Ann Hematol, 2016	388	326	12	49	1
Papaemmanuil et al.[7], N Engl J Med, 2016	1540	1353	152	33	2
**Total (n)**	**1928**	**1679**	**164**	**82**	**3**
***TET2*^{WT} cases (n) (%)**	**1843**	**1679 (91.1%)**	**164 (8.9%)**	–	–
***TET2*^{MUT} cases (n) (%)**	**85**	–	–	**82 (96.5%)***	**3 (3.5%)***

* P=0.0491 (one-tailed Wilson/Brown Binominal test)

Supplemental table 2c: Overlap of *TET2*^{MUT} with *WT1*^{MUT} in *CEBPA*^{DM} AML cases

Cohort	*CEBPA*^{DM} total cases (n)	*TET2*^{WT} *WT1*^{WT} (n)	*TET2*^{WT} *WT1*^{MUT} (n)	*TET2*^{MUT} *WT1*^{WT} (n)	*TET2*^{MUT} *WT1*^{MUT} (n)
Taube et al.[2], Blood, 2022	131	78	21	***29***	3
Zhang et al.[3], Cancer Gene Ther, 2019	76	51	21	2	2
Konstandin et al.[5], Blood Adv, 2018	48	25	3	16	4
Ahn et al.[6], Ann Hematol, 2016	50	39	7	4	0
Papaemmanuil et al.[7], N Engl J Med, 2016	66	40	21	5	0
Fasan et al.[8], Leukemia, 2014	89	48	10	28	3
**Total (n)**	**460**	**281**	**83**	**84**	**12**
***TET2*^{WT} cases (%)**	**364**	**281 (77.2%)**	**83 (22.8%)**	–	–
***TET2*^{MUT} cases (%)**	**96**	–	–	**84 (87.5%)****	**12 (12.5%)****

** P=0.0081 (one-tailed Wilson/Brown Binominal test)

Supplemental table 2d: Overlap of *TET2*^{MUT} with *CSF3R*^{MUT} in *CEBPA*^{DM} AML cases

Cohort	*CEBPA*^{DM} total cases (n)	*TET2*^{WT} *CSF3R*^{WT} (n)	*TET2*^{WT} *CSF3R*^{MUT} (n)	*TET2*^{MUT} *CSF3R*^{WT} (n)	*TET2*^{MUT} *CSF3R*^{MUT} (n)
Taube et al.[2], Blood, 2022	131	96	3	32	0
Zhang et al.[3], Cancer Gene Ther, 2019	76	62	10	4	0
Konstandin et al.[5], Blood Adv, 2018	48	26	2	17	3
Ahn et al.[6], Ann Hematol, 2016	-	N.D.	N.D.	N.D.	N.D.
Papaemmanuil et al.[7], N Engl J Med, 2016	-	N.D.	N.D.	N.D.	N.D.
Fasan et al.[8], Leukemia, 2014	-	N.D.	N.D.	N.D.	N.D.
**Total (n)**	**255**	**184**	**15**	**53**	**3**
***TET2*^{WT} cases (%)**	**199**	**184 (92.5%)**	**15 (7.5%)**	–	–
***TET2*^{MUT} cases (%)**	**56**	–	–	**53 (94.6%)$^{n.s.}$**	**3 (5.4%)$^{n.s.}$**

N.D. not determined; $^{n.s.}$ P=0.3867 (one-tailed Wilson/Brown Binominal test)

Supplemental table 2e: Overlap of *TET2*^{MUT} with *ASXL1*^{MUT} in *CEBPA*^{DM} AML cases

Cohort	*CEBPA*^{DM} total cases (n)	*TET2*^{WT} *ASXL1*^{WT} (n)	*TET2*^{WT} *ASXL1*^{MUT} (n)	*TET2*^{MUT} *ASXL1*^{WT} (n)	*TET2*^{MUT} *ASXL1*^{MUT} (n)
Taube et al.[2], Blood, 2022	131	98	1	28	4
Zhang et al.[3], Cancer Gene Ther, 2019	76	72	0	4	0
Konstandin et al.[5], Blood Adv, 2018	48	28	0	17	3
Ahn et al.[6], Ann Hematol, 2016	50	42	4	4	0
Papaemmanuil et al.[7], N Engl J Med, 2016	66	61	0	5	0
Fasan et al.[8], Leukemia, 2014	89	47	11	26	5
**Total (n)**	**460**	**348**	**16**	**84**	**12**
***TET2*^{WT} cases (%)**	**364**	**348 (95.6%)**	**16 (4.4%)**	_	_
***TET2*^{MUT} cases (%)**	**96**	_	_	**84 (87.5%)****	**12 (12.5%)****

** P=0.0011 (one-tailed Wilson/Brown Binominal test)

Supplemental table 3: CEBPA-mutated patients included in survival analysis

	***CEBPA*^{DM}*TET2*^{WT}**	***CEBPA*^{DM}*TET2*^{MUT}**
Number of patients (n)	84	35
Age at diagnosis, Median [25th –75th percentile] (years)	54 [37-67]	73 [61-88]
Female sex (%)	56.0	40.0
Survival, Median (years)	Undefined	1.84
Hazard ratio [95% CI] (logrank)	0.36 [0.18-0.75]	2.74 [1.34-5.61]

Supplemental table 4: CEBPA-mutated patients included in gene expression analysis (Beat AML study[9])

Patient ID	Sex	Age (years)	*CEBPA*^{MUT} (VAF)	*TET2*^{MUT} (VAF)	*GATA2*^{MUT} (VAF)
AML_OHSU_2018_757	Female	32	Y108* (0.28)	-	-
AML_OHSU_2018_1049	Male	78	D80Sfs*78 (0.28)	-	Y322N (0.33)
AML_OHSU_2018_1159	Male	31	A47Cfs*61 (0.30) V314_L315dup (0.19)	-	-
AML_OHSU_2018_1370	Male	63	F82Sfs*78 (0.26) G340Cfs*84 (0.20)	M695Nfs*17 (0.30) Q1327* (0.26)	
AML_OHSU_2018_1396	Male	60	Q83Hfs*27 (0.20)	-	-
AML_OHSU_2018_1431	Male	55	K304_Q305insL (0.28) F82Lfs*28 (0.18)	-	N317H (0.22)
AML_OHSU_2018_1754	Male	77	L81Rfs*72 (0.40)	M823* (0.33)	-
AML_OHSU_2018_1848	Male	61	A30Pfs*130 (0.30)	-	-
AML_OHSU_2018_2073	Male	85	F77Sfs*83 (0.22)	-	-
AML_OHSU_2018_2113	Male	74	Y108* (0.19)	A1158V (0.19)	-
AML_OHSU_2018_2116	Female	10	T98Hfs*10 (0.32)	-	A318V (0.34)
AML_OHSU_2018_2311	Male	78	Q330* (0.26)	X1395_splice (0.35) C1289Tfs*75 (0.31)	-
AML_OHSU_2018_2322	Male	40	K313dup (0.27) S28Gfs*81 (0.24)	-	G320D (0.11)
AML_OHSU_2018_2630	Female	46	T98Hfs*10 (0.35) R297P (0.27)	Q758* (0.30)	-
AML_OHSU_2018_2713	Male	47	R35Gfs*125	-	-
AML_OHSU_2018_2737	Male	20	K90Efs*18 (0.34)	-	-

Supplemental table 5: sgRNA sequences

sgRNA	Sequence
sgTet2 (crRNA)	AAGGAGGCTGAAGAACAAGA
sgGata2 (crRNA)	TCTCTGGCGACGAGATGGCA
sgG2DHE_A	TGACGTAGCAAGCTGAGCGC
sgG2DHE_1	AGCCATCAGGCCCTGCAGCA
sgG2DHE_2	GTATCCTGATGTGGTAAAGA
sgG2DHE_3	TGCATGAATTCCGGTCTCAA
sgG2DHE_4	GCTGTGCGGTGGGCAGAACG
sgG2DHE_5	CACCCCCCAGGCAGTGGACA
sgG2DHE_6	ATCATCTGCCAGCAGAGGCC
sgG2DHE_B	GAGCGACCTTTGCAGCAGCA

Supplemental table 6: PCR and qPCR primers

Target	Primer 1 (5' to 3')	Primer 2 (5' to 3')	Primer 3 (5' to 3')
Cebpa wt-fl	GACTCCATGGGGGAGTTAGAG	GCCTTGGAAAGTCACAGGAG	-
Cebpa fl-ko	CCGCGGCTCCACCTCGTAGAAGTCG	CCACTCACCGCCTTGGAAAGTCACA	GTCCTGCAGCCAGGCAGTGTC
Cebpa p30 ki	CCGACTTCTACGAGGTGGAG	CTGTCGGCTGTGCTGGAAGA	-
Tet2 wt-fl-ko	GGCAGAGGCATGTTGAATGA	TAGACAAGCCCTGCAAGCAA	GTGTCCCACGGTTACACACG
Mx1-Cre	GCCTGCATTACCGGTCGATGCAACGA	GTGGCAGATGGCGCGGCAACACCATT	-
R26-Cre-ER	AAAGTCGCTCTGAGTTGTTAT	GGAGCGGGAGAAATGGATATG	CCTGATCCTGGCAATTTCG
R26-rtTA	AAAGTCGCTCTGAGTTGTTAT	GGAGCGGGAGAAATGGATATG	GCGAAGAGTTTGTCCTCAACC
MLL-AF9 ki	CTAGATCTCGAAGGATCTGGAG	ATACTTTCTCGGCAGGAGCA	-
miR30	CAGAAGGCTCGAGAAGGTATATTGCTGTTGACAGTGAGCG	CTAAAGTAGCCCCTTGAATTCCGAGGCAGTAGGCA	-
Gata2 mRNA (for Cebpa$^{p30/-}$)	GCAGAGAAGCAAGGCTCGC	CAGTTGACACACTCCCGGC	-
Gata2 mRNA (for Cebpa$^{p30/p30}$)	ACAGGCCACTGACCATGAAG	AAGGGCGGTGACTTCTCTTG	
Gata2 V1 mRNA	CCGCTGCGAGTGGCC	GCCCGGATGGTGCGA	-
Gata2 V2 mRNA	GCCGCAGTCGGGCC	CTGCTCAGGCGCCACCT	-
Actb mRNA	AAGGAGATTACTGCTCTGGCTCCTA	ACTCATCGTACTCCTGCTTGCTGAT	-
Gapdh mRNA	AGAAGGTGGTGAAGCAGGCAT	CGGCATCGAAGGTGGAAGAGT	-
Gata2 V2 Bisulfite conv. DNA	GGAATTTTTTTAGTGGGATTTTAATAAG	ATACAATTTACTTACAATTTATCAACCC	-
Gata2 (TIDE genotyping)	TTTCCGGGTAACTTGCTGCT	TCAGGTGGTGAAGTGTCTGC	-
Tet2 (TIDE genotyping)	TGACTTTTCAGGGCTCGGTG	CGAGATCCCTCAACCATCGC	-
G2DHE (for ChIP-qPCR)	AATTCTGGTCAACCGCAAGC	TCTCGCATCCGTTACTTGCC	

Supplemental table 7: ShRNA sequences

ShRNA	Identifier Mission® shRNA	Sequence of 97-mer (5' to 3')
ShGata2 A	TRCN0000085421	TGCTGTTGACAGTGAGCGACCTGCAACACACCACCCGATATAGTGAAGCCACAGATGTATATCGGGTGGTGTGTTGCAGGGTGCCTACTGCCTCGGA
ShGata2 B	TRCN0000085419	TGCTGTTGACAGTGAGCGACTCTACTACAAGCTGCACAATTAGTGAAGCCACAGATGTAATTGTGCAGCTTGTAGTAGAGGTGCCTACTGCCTCGGA
ShGata2 C	TRCN0000085418	TGCTGTTGACAGTGAGCGACCCTGTAAATACAACCTTCTTTAGTGAAGCCACAGATGTAAAGAAGGTTGTATTTACAGGGGTGCCTACTGCCTCGGA
ShGata2 D	TRCN0000321390	TGCTGTTGACAGTGAGCGACCGCCATTACTGTGAATATTTTAGTGAAGCCACAGATGTAAAATATTCACAGTAATGGCGGGTGCCTACTGCCTCGGA

Supplemental table 8: **Antibodies for flow cytometry and FACS**

Antigen	Name	RRID	Manufacturer	Catalogue #	Fluorophore	Clone	Dilution
CD45.2	PE Mouse Anti-Mouse CD45.2	AB_1727493	BD Pharmingen™ (BD Bioscience)	560695	PE	104	1:200
CD3e	CD3e Monoclonal Antibody	AB_468690	eBioscience™ (Thermo Fisher Scientific)	15-0031-82	PE-Cy5	145-2C11	1:400
CD45R/B220	CD45R (B220) Monoclonal Antibody	AB_468756	eBioscience™ (Thermo Fisher Scientific)	15-0452-83	PE-Cy5	RA3-6B2	1:400
Ly76/Ter119	TER-119 Monoclonal Antibody	AB_468809	eBioscience™ (Thermo Fisher Scientific)	15-5921-81	PE-Cy5	TER-119	1:400
Ly76/Ter119	TER-119 Monoclonal Antibody	AB_469661	eBioscience™ (Thermo Fisher Scientific)	25-5921-82	PE-Cy7	TER-119	1:400
Ly6G+Ly6C/Gr1	APC Rat Anti-Mouse Ly-6G and Ly-6C	AB_398532	BD Pharmingen™ (BD Bioscience)	553129	APC	RB6-8C5	1:400
Ly6G+Ly6C/Gr1	Ly-6G/Ly-6C Monoclonal Antibody	AB_468813	eBioscience™ (Thermo Fisher Scientific)	15-5931-82	PE-Cy5	RB6-8C5	1:400
CD11b/Mac1	FITC Rat Anti-CD11b	AB_396679	BD Pharmingen™ (BD Bioscience)	553310	FITC	M1/70	1:800
CD11b/Mac1	PE/Cyanine5 anti-mouse/human CD11b Antibody	AB_312793	Biolegend™ (NordicBiosite)	101210	PE-Cy5	M1/70	1:800
CD117/c-Kit	CD117 (c-Kit) Monoclonal Antibody	AB_1272177	eBioscience™ (Thermo Fisher Scientific)	47-1171-82	APC eF780	2B8	1:200
CD41a	CD41a Monoclonal Antibody	AB_763481	eBioscience™ (Thermo Fisher Scientific)	11-0411-82	FITC	eBioMWReg30	1:200
Ly6A+Ly6E/Sca-1	Ly-6A/E (Sca-1) Monoclonal Antibody	AB_914372	eBioscience™ (Thermo Fisher Scientific)	45-5981-82	PerCp-Cy5.5	D7	1:200
CD16+CD32/FcgRII/III	CD16/CD32 Monoclonal Antibody	AB_493994	eBioscience™ (Thermo Fisher Scientific)	56-0161-82	Alexa Fluor 700	93	1:100

SUPPLEMENTAL REFERENCES

1 Jakobsen, J. S. *et al.* Mutant CEBPA directly drives the expression of the targetable tumor-promoting factor CD73 in AML. *Sci Adv* **5**, eaaw4304, doi:10.1126/sciadv.aaw4304 (2019).

2 Taube, F. *et al.* CEBPA mutations in 4708 patients with acute myeloid leukemia: differential impact of bZIP and TAD mutations on outcome. *Blood* **139**, 87-103, doi:10.1182/blood.2020009680 (2022).

3 Zhang, Y. *et al.* Companion gene mutations and their clinical significance in AML with double mutant CEBPA. *Cancer Gene Ther*, doi:10.1038/s41417-019-0133-7 (2019).

4 Su, L. *et al.* Mutational spectrum of acute myeloid leukemia patients with double CEBPA mutations based on next-generation sequencing and its prognostic significance. *Oncotarget* **9**, 24970-24979, doi:10.18632/oncotarget.23873 (2018).

5 Konstandin, N. P. *et al.* Genetic heterogeneity of cytogenetically normal AML with mutations of CEBPA. *Blood Adv* **2**, 2724-2731, doi:10.1182/bloodadvances.2018016840 (2018).

6 Ahn, J. S. *et al.* Normal karyotype acute myeloid leukemia patients with CEBPA double mutation have a favorable prognosis but no survival benefit from allogeneic stem cell transplant. *Ann Hematol* **95**, 301-310, doi:10.1007/s00277-015-2540-7 (2016).

7 Papaemmanuil, E. *et al.* Genomic Classification and Prognosis in Acute Myeloid Leukemia. *N Engl J Med* **374**, 2209-2221, doi:10.1056/NEJMoa1516192 (2016).

8 Fasan, A. *et al.* The role of different genetic subtypes of CEBPA mutated AML. *Leukemia* **28**, 794-803, doi:10.1038/leu.2013.273 (2014).

9 Tyner, J. W. *et al.* Functional genomic landscape of acute myeloid leukaemia. *Nature* **562**, 526-531, doi:10.1038/s41586-018-0623-z (2018).
