# Supplementary material for: TET2 lesions enhance the aggressiveness of CEBPA-mutant acute myeloid leukemia by rebalancing GATA2 expression
Source: Nat Commun. 2023 Oct 4;14:6185. doi: 10.1038/s41467-023-41927-x (PMC10550934; doi:10.1038/s41467-023-41927-x)
Supplement: Supplementary file 3 — Reporting Summary [file 41467_2023_41927_MOESM3_ESM.pdf]

nature portfolio

Corresponding author(s): Florian Grebien
Bo Porse

Last updated by author(s): Sep 13, 2023

# Reporting Summary

Nature Portfolio wishes to improve the reproducibility of the work that we publish. This form provides structure for consistency and transparency in reporting. For further information on Nature Portfolio policies, see our Editorial Policies and the Editorial Policy Checklist.

## Statistics

For all statistical analyses, confirm that the following items are present in the figure legend, table legend, main text, or Methods section.

| n/a | Confirmed | |
|---|---|---|
| ☐ | ☒ | The exact sample size ($n$) for each experimental group/condition, given as a discrete number and unit of measurement |
| ☐ | ☒ | A statement on whether measurements were taken from distinct samples or whether the same sample was measured repeatedly |
| ☐ | ☒ | The statistical test(s) used AND whether they are one- or two-sided<br>*Only common tests should be described solely by name; describe more complex techniques in the Methods section.* |
| ☐ | ☒ | A description of all covariates tested |
| ☐ | ☒ | A description of any assumptions or corrections, such as tests of normality and adjustment for multiple comparisons |
| ☐ | ☒ | A full description of the statistical parameters including central tendency (e.g. means) or other basic estimates (e.g. regression coefficient) AND variation (e.g. standard deviation) or associated estimates of uncertainty (e.g. confidence intervals) |
| ☐ | ☒ | For null hypothesis testing, the test statistic (e.g. $F$, $t$, $r$) with confidence intervals, effect sizes, degrees of freedom and $P$ value noted<br>*Give P values as exact values whenever suitable.* |
| ☒ | ☐ | For Bayesian analysis, information on the choice of priors and Markov chain Monte Carlo settings |
| ☒ | ☐ | For hierarchical and complex designs, identification of the appropriate level for tests and full reporting of outcomes |
| ☒ | ☐ | Estimates of effect sizes (e.g. Cohen's $d$, Pearson's $r$), indicating how they were calculated |

*Our web collection on statistics for biologists contains articles on many of the points above.*

## Software and code

Policy information about availability of computer code

| | |
|---|---|
| Data collection | Mutation and expression data from the BeatAML study was acquired at http://vizome.org/aml or https://www.cbioportal.org/, and downloaded using Synapse.<br>Sequencing data in this study was obtained on a HiSeq4A or NextSeq 500.<br>Flow cytometry data was obtained on a BD FACSAriaTM III or a BD LSR IITM (BD Bioscience). |
| Data analysis | Bio-informatics analyses of RNA-seq data:<br>Quality check: FastQC (v. 0.11.4)<br>Preprocessing: PRINSEQ-lite (version 0.20.4)<br>Read alignment: BWA (version 0.7.15)<br>Differential gene expression analysis: DESeq2 (version 1.22.2).<br>or<br>Read processing: the bcbio RNA-seq pipeline (https://github.com/bcbio/bcbio-nextgen) and the bcbioRNASeq R package (https://github.com/hbc/bcbioRNASeq).<br>Transcript abundance: Salmon (v. 0.12.0)<br>Summary to gene level and import into R: tximport (v. 1.10.1)<br>Differential gene expression analysis: DESeq2 (v. 1.22.2).<br><br>Gene expression analysis between different data sets: edgeR (v. 3.32.1)<br><br>GSEA: GSEA software (v. 4.1.0). |

Bioinformatics analyses of ATAC-seq data:
Quality check: FastQC (version 0.11.4)
Preprocessing: PRINSEQ-lite (version 0.20.4)
Alignment: BWA (version 0.7.15)
Post processing: SAMtools (version 1.4)
Peaks calling: MACS2 (version 2.1.0)
Differential analysis: DiffBind (version 2.16.0)
Region enrichment analysis: GREAT (version 4.0.4)
Motif analysis: HOMER (v. 4.11)

Bio-informatics analyses of WGBS data:
Read trimming and filtered: Trim Galore (v. 0.4.3)
Quality assessment FastQC (v. 0.11.7)
Alignment: Bismark (v. 0.19.1) and Bowtie2 (v. 2.2.8)
Visualization: deepTools (v. 3.1.3) and UCSC's bedGraphToBigWig (v. 4)

Bioinformatic analyses of ChIP-seq data:
Quality checking: FastQC (version 0.11.4)
Preprocessed with PRINSEQ-lite (version 0.20.4)
Alignment: BWA (version 0.7.15 or 0.7.17)
Processing: SAMtools (version 1.4 or 1.13)
Normalization: Deeptools (version 2.5.0 or 3.5.1)
Peak calling: MACS2 (version 2.1.0)
Annotation: HOMER (version 4.9.1)
Visualization:IGV (version 2.6.0)
or
Mapping: Bowtie2 (v. 2.3.4.3) and MACS2 (v. 2.1.0.20151222).
Normalization: deepTools (v. 3.3.1).
Read count and differential analysis: bedtools (v. 2.30.0) and DESeq2 (v. 1.30.1)

Flow cytometry analysis:
FlowJo software (v9)

For manuscripts utilizing custom algorithms or software that are central to the research but not yet described in published literature, software must be made available to editors and reviewers. We strongly encourage code deposition in a community repository (e.g. GitHub). See the Nature Portfolio guidelines for submitting code & software for further information.

## Data

Policy information about availability of data

All manuscripts must include a data availability statement. This statement should provide the following information, where applicable:

- Accession codes, unique identifiers, or web links for publicly available datasets
- A description of any restrictions on data availability
- For clinical datasets or third party data, please ensure that the statement adheres to our policy

The data generated in this study is publicly available in Gene Expression Omnibus (GEO) under accession numbers GSE214224 (RNA-seq , TET2-ChIP and ATAC-seq in vitro) and GSE213864 (RNA-seq and WGBS in vivo), and within the article and its supplementary files.

Publically avaliable data sets used in this study:
CEBPA and H3K27Ac ChIP-seq from myeloid progenitor cell model for p30-driven AML is available under GSE158727 (Heyes et al.).
CEBPA ChIP-seq from mouse Cebpa+/+ or Cebpap30/p30 GMPs is available under GSE118963 (Jakobsen et al.).
RNA-seq data from Cebpap30/p30 AML are available under GSE118963 (Jakobsen et al.) and GSE141477 (Trempenau et al.).
Patient data analyzed in this study were from the Beat AML study (Tyner et al.; accessed through cBioPortal (https://www.cbioportal.org/) or Vizome (http://www.vizome.org/)) or from published cohort studies.

Source data are provided with this paper.

## Human research participants

Policy information about studies involving human research participants and Sex and Gender in Research.

| | |
|---|---|
| Reporting on sex and gender | Patient data in this study has not been stratified based on sex nor gender. |
| Population characteristics | All patents included in the retrospective survival analysis were diagnosed with AML, and mutational status was the only factor taken into consideration. The cohort included 119 patients with a median age at diagnosis of 58.30 years (39.90–73.30 years 25th–75th percentile) and the sex distribution was 48.7% males and 51.3% females. The only parameters available to us were mutational status, age, and sex. |

| | |
|---|---|
| Recruitment | Data on mutational status and survival of CEBPA-mutant patients was provided from the MLL Munich Leukemia Laboratory, Munich, Germany, which provides a nationwide diagnostic service in Germany. |
| Ethics oversight | All patients gave written informed consent for the use of data for scientific evaluations. The study was approved by the Internal Review Board and by the Bavarian Ethics Committee, the Bavarian State Medical Association (Bayerische Landesärztekammer) with the number 05117. The study adhered to the tenets of the Declaration of Helsinki. |

Note that full information on the approval of the study protocol must also be provided in the manuscript.

# Field-specific reporting

Please select the one below that is the best fit for your research. If you are not sure, read the appropriate sections before making your selection.

☒ Life sciences ☐ Behavioural & social sciences ☐ Ecological, evolutionary & environmental sciences

For a reference copy of the document with all sections, see nature.com/documents/nr-reporting-summary-flat.pdf

# Life sciences study design

All studies must disclose on these points even when the disclosure is negative.

| | |
|---|---|
| Sample size | No statistical method was applied to predetermine sample sizes. Sample sizes were determined based on previous studies performed in the labs using similar techniques (e.g., Schmidt et al. 2019, Heyes et al. 2020, D'Altri et al. 2021, Trempenau et al. 2023, and Jakobsen et al 2019) as well as the consensus in the field.<br>For the retrospective study of survival and differential gene expression of CEBPAdm AML, the maximum number of obtainable samples were analyzed. |
| Data exclusions | For in vivo, ex vivo and in vitro experiments, data were not excluded from analysis.<br><br>For RNA expression analysis of data from the BeatAML data set, we excluded patients that had co-occurring mutation(s) in WT1 or IDH1/2 since these have been shown to interfere with TET2 function as well as two patients with low CEBPA variant allele frequency (VAF).<br><br>For survival analysis of CEBPAdm AML, all available data was included in the analysis. |
| Replication | The number of biological replicates (i.e., number of mice, number individually experiments with a cell line, and number of patients) are given in the figure legends.<br><br>For patient data we had access to one cohort of patients with enough patients for evaluation of effects on survival. Patient data evaluating co-occurring mutations were derived from 7 separate cohorts. Expression data were based on all available samples in the publicly available Beat AML study.<br><br>In vitro data were generated using several individual clones of each mutated primary cell line (n>3) and/or cell lines were generated from 2–3 separate mice. For in vitro targeting of the Gata2 enhancer, 12 sgRNA pairs were tested in 2 independent experiments.<br>RNA and ATAC seq data were generated from n>4 individual clones.<br><br>In vivo experiments included 2–4 separate donor mice each represented by >3 recipient mice both for evaluating leukemic initiation and leukemia onset in secondary recipients. Response to therapy was evaluated for leukemias from 2–3 secondary leukemic clones generated form separate primary-through-secondary leukemias and survival was evaluated in one of these clones by response in >4 recipient mice.<br>RNAseq and WGBS data were generated from samples from n=2–3 mice. The shRNA knockdown of Gata2 was evaluated in >3 recipients per construct and re-evaluated in an independent experiment (the non-monotonic correlation between knockdown levels and competitiveness were somewhat counterintuitive to us) and the validation was successful. |
| Randomization | The retrospective study involving patient data was not randomized as no intervention was done, hence randomization is inherently impossible. The samples were stratified based on mutation status or CEBPA levels.<br>Recipient mice were randomized to receive control and test leukemic cells by random picking by a technician not involved in the study. For survival analysis, individual mice were shuffled between cages. |
| Blinding | The data collection and analysis were not blinded. Blinding was not feasible since the investigators performed both experimental procedures and analysis of data. |

# Reporting for specific materials, systems and methods

We require information from authors about some types of materials, experimental systems and methods used in many studies. Here, indicate whether each material, system or method listed is relevant to your study. If you are not sure if a list item applies to your research, read the appropriate section before selecting a response.

## Materials & experimental systems

| n/a | Involved in the study |
|---|---|
| ☐ | ☒ Antibodies |
| ☐ | ☒ Eukaryotic cell lines |
| ☒ | ☐ Palaeontology and archaeology |
| ☐ | ☒ Animals and other organisms |
| ☒ | ☐ Clinical data |
| ☒ | ☐ Dual use research of concern |

## Methods

| n/a | Involved in the study |
|---|---|
| ☐ | ☒ ChIP-seq |
| ☐ | ☒ Flow cytometry |
| ☒ | ☐ MRI-based neuroimaging |

# Antibodies

Antibodies used

Western blotting:
anti-TET2, Santa Cruz, sc-398535, 1:100
anti-HSC70, Santa Cruz, sc-7298, 1:10000

Chromatin immunoprecipitation:
anti-C/EBPα, Santa Cruz, sc-9314, 1:60
anti-TET2, Cell signaling technology, 36449, 1:50

Flow cytometry / FACS:
Antigen, Name, Manufacturer, Catalogue #, Fluorophore, Clone, Dilution
CD45.2, PE Mouse Anti-Mouse CD45.2, BD Pharmingen™ (BD Bioscience), 560695, PE, 104, 1:200
CD3e, CD3e Monoclonal Antibody, eBioscience™ (Thermo Fisher Scientific), 15-0031-82, PE-Cy5, 145-2C11, 1:400
CD45R/B220, CD45R (B220) Monoclonal Antibody, eBioscience™ (Thermo Fisher Scientific), 15-0452-83, PE-Cy5, RA3-6B2, 1:400
Ly76/Ter119, TER-119 Monoclonal Antibody, eBioscience™ (Thermo Fisher Scientific), 15-5921-81, PE-Cy5, TER-119, 1:400
Ly76/Ter119, TER-119 Monoclonal Antibody, eBioscience™ (Thermo Fisher Scientific), 25-5921-82, PE-Cy7, TER-119, 1:400
Ly6G+Ly6C/Gr1, APC Rat Anti-Mouse Ly-6G and Ly-6C, BD Pharmingen™ (BD Bioscience), 553129, APC, RB6-8C5, 1:400
Ly6G+Ly6C/Gr1, Ly-6G/Ly-6C Monoclonal Antibody, eBioscience™ (Thermo Fisher Scientific), 15-5931-82, PE-Cy5, RB6-8C5, 1:400
CD11b/Mac1, FITC Rat Anti-CD11b, BD Pharmingen™ (BD Bioscience), 553310, FITC, M1/70, 1:800
CD11b/Mac1, PE/Cyanine5 anti-mouse/human CD11b Antibody, Biolegend™ (NordicBiosite), 101210, PE-Cy5, M1/70, 1:800
CD117/c-Kit, CD117 (c-Kit) Monoclonal Antibody, eBioscience™ (Thermo Fisher Scientific), 47-1171-82, APC, eF780, 2B8, 1:200
CD41a, CD41a Monoclonal Antibody, eBioscience™ (Thermo Fisher Scientific), 11-0411-82, FITC, eBioMWReg30, 1:200
Ly6A+Ly6E/Sca-1, Ly-6A/E (Sca-1) Monoclonal Antibody, eBioscience™ (Thermo Fisher Scientific), 45-5981-82, PerCp-Cy5.5, D7, 1:200
CD16+CD32/FcgRII/III, CD16/CD32 Monoclonal Antibody, eBioscience™ (Thermo Fisher Scientific), 56-0161-82, Alexa Fluor 700, 93, 1:100

Magnetic separation:
mouse CD117 MicroBeads, 1:10 130-091-224, Miltenyi Biotec

Immuno-histochemistry:
anti-Ki67 antibody, 1:50, ab16667, Abcam

Validation

The commercial antibody used have been validated by the manufacturers:
anti-TET2 sc-398535 and anti-HSC70 sc-7298 are validated for WB on mouse cells by manufacturer.
anti-CEBPA sc-9314 and anti-TET2 #36449 are validated for ChIP by the manufacturer.
All antibodies used for flow cytometry and FACS are validated for these methods on mouse cells by the manufacturers.
CD117 Microbeads 130-091-224 are validated for use on mouse cells by the manufacturer.
anti-Ki67 ab16667 is validated for IHC on mouse cells by the manufacturer.

# Eukaryotic cell lines

Policy information about cell lines and Sex and Gender in Research

Cell line source(s)

Establishment of Cebpa-mutant cell line: Primary murine fetal liver cells from a male Cebpap30/p30 mouse were cultivated in DMEM plus 10% FCS plus SCF, IL-6, and IL-3. For colony assays, 1×104 cells were plated in complete methylcellulose medium (MethoCult M3434). The Cebpap30/p30 cell line was established by picking single cell clones after the 6th round of re-plating and continuous liquid culture in the presence of SCF, IL-3 and IL-6 for 4 weeks.

Establishment of ex vivo Cebpa-/p30Tet2+/+ and Cebpa-/p30Tet2-/- lines: Thawed cryo-preserved cells from female primary AML were cultured in Lonza X-VivoTM 15 cell medium (#BE02-060Q Thermo Fisher Scientific) supplemented with Bovine Serum Albumin in Iscove's MDM (10%; #09300 StemcellTM Technologies), Penicillin-Streptomycin (1%; #15140122 Gibco), B-mercaptoethanol (0.1 mM; #31350010 Gibco), L-glutamine (2 mM; #25030149 Gibco), and cytokines h-IL-6 (50 ng/ml; #130-093-032 Miltenyi Biotec), m-SCF (50 ng/μl; #250-03 Peprotech), m-IL-3 (10 ng/ml; #213-13 Peprotech), and m-GM-CSF (10 ng/ml; #315-03 Peprotech). Two clones of each genotype continued to expand beyond 40 days and withstood freeze-thawing, and these clones have been used for further experiments.

| | |
|---|---|
| | Establishment of ex vivo iMLL-AF9+Cebpafl/flR26-CreER+ lines: Sorted GMPs from female iMLL-AF9+Cebpafl/flR26CreER+ mice, were cultured in MethoCult (M3434; #03434, Stemcell technologies) supplemented with doxycycline (1 µg/ml; #D9891 Sigma-Aldrich) for three replatings to induce expression of the MLL-fusion protein.<br><br>Retro-virus for shRNA knockdown was generated by transfection of Phenix-Eco cells (CVCL_H171). |
| Authentication | The cell lines were not authenticated. |
| Mycoplasma contamination | Cell lines were tested for mycoplasma contamination twice yearly and have tested negative. |
| Commonly misidentified lines (See ICLAC register) | No commonly misidentified lines were used in this study. |

## Animals and other research organisms

Policy information about studies involving animals; ARRIVE guidelines recommended for reporting animal research, and Sex and Gender in Research

| | |
|---|---|
| Laboratory animals | Species: Mouse (Mus Musculus)<br>Strains: C57BL/6J (donor mice) or C57BL/6J.SJL (recipient mice)<br>Genotypes: Cebpa+/fl, Cebpafl/p30, Cebpap30/p30, Tet2+/+, Tet2fl/fl, Mx1-Cre+, and combination thereof.<br>Sex: Female<br>Age: 10-12 weeks of age at experimental start<br>The mice were housed in a temperature- and humidity-controlled room with a 06:00–18:00 h light cycle and fed a standard chow diet and tap water ad libitum. |
| Wild animals | No wild animals were used in this study. |
| Reporting on sex | Female mice were used in this study. |
| Field-collected samples | No field-collected samples were used in this study. |
| Ethics oversight | The Danish Animal Ethical Committee |

Note that full information on the approval of the study protocol must also be provided in the manuscript.

## ChIP-seq

### Data deposition

- [x] Confirm that both raw and final processed data have been deposited in a public database such as GEO.
- [x] Confirm that you have deposited or provided access to graph files (e.g. BED files) for the called peaks.

| | |
|---|---|
| Data access links<br>*May remain private before publication.* | We have deposited our data in the GEO repository:<br>https://www.ncbi.nlm.nih.gov/geo/query/acc.cgi?acc=GSE213864<br>https://www.ncbi.nlm.nih.gov/geo/query/acc.cgi?acc=GSE214224 |
| Files in database submission | GSE214224 (RNA-seq , TET2-ChIP and ATAC-seq in vitro)<br>GSE213864 (RNA-seq and WGBS in vivo) |
| Genome browser session (e.g. UCSC) | Not applicable |

### Methodology

| | |
|---|---|
| Replicates | Three biological replicates of TET2 ChIP in Cebpap30/p30 cells and one Cebpap30/p30 input sample were sequenced. |
| Sequencing depth | Files were paired-end (2x 100 bp).<br>File (Reads pairs in,;Reads in; Reads mapped; %_mapped;not mapped)<br>Cebpap30/p30 #1 (53,287,894; 106,575,788 ;89,111,475 ;83.61% ;17,464,313)<br>Cebpap30/p30 #2 (69,680,653; 139,361,306; 118,852,334 ;85.28%; 20,508,972)<br>Cebpap30/p30 #3 (52,051,629; 104,103,258; 91,808,620; 88.19%; 12,294,638)<br>Cebpap30/p30 INPUT (57.286.886;114.573.772;98.381.882;85,87% ;16.191.890) |
| Antibodies | anti-TET2, Cell signaling technology, 36449 |
| Peak calling parameters | Mapping parameters: bwa mem -t $THREADS $mmREF $fastq1 $fastq2 > $WRKPREFIX.sam<br>Peak calling parameters: python2 macs2 callpeak -f BAMPE -t $FILE -c $INPUT -g mm -q 0.05 -n $OUTFILE 2> $LOG<br><br>bwa mem -t 16 mmREF shRen1_1.fastq shRen1_2.fastq > shRen1.sam |

```
bwa mem -t 16 mmREF shRen2_1.fastq shRen2_2.fastq > shRen2.sam
bwa mem -t 16 mmREF shRen3_1.fastq shRen3_2.fastq > shRen3.sam
bwa mem -t 16 mmREF shRenINPUT_1.fastq shRenINPUT_2.fastq > shRenINPUT.sam
python2 macs2 callpeak -f BAMPE -t shRen1.bam -c shRenINPUT.bam -g mm -q 0.05 -n shRen1 2> log/shRen2.log
python2 macs2 callpeak -f BAMPE -t shRen2.bam -c shRenINPUT.bam -g mm -q 0.05 -n shRen2 2> log/shRen2.log
python2 macs2 callpeak -f BAMPE -t shRen3.bam -c shRenINPUT.bam -g mm -q 0.05 -n shRen3 2> log/shRen2.log
```

Data quality

Number of peaks below FDR 5% and above 5-fold enrichment: shRen1 1743 , shRen2 940 , shRen3 847

Software

Sequencing reads derived from Tet2 ChIP-seq experiment were preprocessed with PRINSEQ-lite and the remaining reads were mapped to the mouse reference genome sequence (mm10) using BWA (version 0.7.17-r1188). The resulting alignments were processed with samtools (version 1.13) and peak calling was done with MACS2 (version 2.1.0.20140616). Aligned read counts were normalized to CPM using the bamCoverage function from deeptools (version 3.5.1).

# Flow Cytometry

## Plots

Confirm that:

- [x] The axis labels state the marker and fluorochrome used (e.g. CD4-FITC).
- [x] The axis scales are clearly visible. Include numbers along axes only for bottom left plot of group (a 'group' is an analysis of identical markers).
- [x] All plots are contour plots with outliers or pseudocolor plots.
- [x] A numerical value for number of cells or percentage (with statistics) is provided.

## Methodology

Sample preparation

To analyze the composition of either freshly isolated or thawed cryopreserved BM and blood, cells were stained with fluorescently labelled antibodies. For blood analysis, 50 µl blood was collected from the facial vein and erythrocytes were lysed with lysing buffer (BD Pharm LyseTM, #555899 BD Bioscience). For BM analysis, cells were collected by crushing tibia, femur, and ilium and filtered through a 50 um filcon cup (#340630 BD Bioscience). Blood or BM cells were washed in PBS with 3% FBS and stained with fluorescently labelled antibodies for 30 min at 4°C. For cryopreserved cells, the cells were counterstained with DAPI (1:10000; #D3571 Invitrogen) to separate out dead cells.

Instrument

BD FACSAria III or a BD LSR II (BD Bioscience)

Software

FlowJo analysis software (Version 9, TreeStar Inc., San Carlos, CA, USA)

Cell population abundance

N.D.

Gating strategy

Cells were gated on FSC and SSC, excluding debris. FSC vs FSH was used to remove duplicates and when applicable, dead cells were excluded using DAPI. Gates defining positive and negative populations for specific markers were set according to fluorescence-minus-one (FMO) controls.

- [x] Tick this box to confirm that a figure exemplifying the gating strategy is provided in the Supplementary Information.
